# [Retracted] Protective effect of erythropoietin against myocardial injury in rats with sepsis and its underlying mechanisms

**DOI:** 10.3892/mmr.2025.13711

**Published:** 2025-10-14

**Authors:** Xinliang Zhang, Shimin Dong, Yanjun Qin, Xiaohua Bian

Mol Med Rep 11: 3317–3329, 2015; DOI: 10.3892/mmr.2015.3155

Following the publication of the above paper, it was drawn to the Editor's attention by a concerned reader that the histological data featured in Fig. 8A of this paper had already appeared in Fig. 3 in an article published by the same research group in the journal *World Journal of Emergency Medicine* in 2013. Subsequently, the Editorial Office performed an independent assessment of the overlapping data between these two papers, and even though additional experiments were performed in the article reported above, including the creation of an alternative sepsis model with Kunming mice and the inclusion of pathological data for the rats in organs other than the heart, it remained the case that Figs. 1–3 of the *World Journal of Emergency Medicine* article and many of the data in Tables 1–3 (albeit in graphical form) were included in the above paper.

Given that almost all of the data featured in the *World Journal of Emergency Medicine* paper had apparently been included in the above article, the Editor of *Molecular Medicine Reports* has decided that this should be retracted on account of these data having been published previously. The authors were asked for an explanation to account for these concerns, but the Editorial Office did not receive a reply. The Editor apologizes for any inconvenience caused to the readership of the Journal.

